# Simultaneous oral administration of *Salmonella* Infantis and *S.* Typhimurium in chicks

**DOI:** 10.1186/s13620-017-0105-x

**Published:** 2017-08-31

**Authors:** Koichi Murakami, Eriko Maeda-Mitani, Daisuke Onozuka, Tamie Noda, Nobuyuki Sera, Hirokazu Kimura, Shuji Fujimoto, Satoshi Murakami

**Affiliations:** 1 Infectious Disease Surveillance Center, National Institute of Infectious Diseases, 4-7-1 Gakuen, Musashi-murayama, Tokyo, 208-0011 Japan; 2 Fukuoka Institute of Health and Environmental Sciences, Mukaizano 39, Dazaifu, Fukuoka, 818-0135 Japan; 3 Department of Health Care Administration and Management, Kyushu University Graduate School of Medical Sciences, 3-1-1 Maidashi, Higashi-ku, Fukuoka, 812-8582 Japan; 4 Department of Health Sciences, Faculty of Medical Sciences, Kyushu University, 3-1-1 Maidashi, Higashi-ku, Fukuoka, 812-8582 Japan; 5 Department of Animal Science, Tokyo University of Agriculture, Atsugi, Kanagawa 243-0034 Japan; 6 Present address: Kitachikugo Office for Health, Human Services, and Environmental Issues, 1642-1 Aikawa-machi Kurume, Fukuoka, 839-0861 Japan

**Keywords:** *Salmonella* infantis, *Salmonella* typhimurium, Chicken, Basic reproductive rate, Oral administration, Chick bowel

## Abstract

**Background:**

To confirm the hypothesis that *Salmonella enterica* subspecies *enterica* serovar (*S.*) Infantis has higher basic reproductive rates in chicks compared with other *Salmonella* serovars, 1-day-old specific-pathogen-free chicks (*n* = 8) were challenged simultaneously with *S.* Infantis and *S.* Typhimurium *per os*. Challenged chicks (Group A) were then housed with non-infected chicks (Group B, *n* = 4) for 6 days (from 2 to 8 days of age). Group B birds were then housed with other non-infected birds (Group C, *n* = 4), which were then transferred to cages containing a further group of untreated chicks (Group D, *n* = 2). A control group consisting of four non-infected chicks was used for comparison. All chickens were humanely sacrificed at 18 days of age, and *Salmonella* from bowel and liver samples were enumerated.

**Results:**

Both serovars were isolated from all groups except the control group. *S.* Typhimurium was isolated at a greater frequency than *S.* Infantis from the bowel samples of chicks from Groups B, C and D, while no differences in colonisation rates were observed between the two serovars in liver samples from Groups B, C and D. *S.* Typhimurium, but not *S.* Infantis, was immunohistochemically detected in the lamina propria of the cecum and rectum in five birds of Group A. Despite the competitive administration, neither of the two serovars completely excluded the other, and no differences were observed in basic reproductive rates between the two serovars.

**Conclusions:**

These findings, together with data from previous studies, suggest that the initial quantitative domination of *S.* Infantis in chicken flocks may explain why this serovar is predominant in broiler chickens.

## Background

Human infections caused by ingestion of chicken meat contaminated with *Salmonella enterica* subsp. *enterica* serovar (*S.*) Infantis are a significant public health concern in many countries, including Japan [1, 2]. Salmonellosis caused by non-typhoidal *Salmonella* serovars occurs fairly frequently worldwide [3]. *S.* Infantis is a major non-typhoidal *Salmonella* serovar in Japan, and is the predominant *Salmonella* contaminant of chicken meat. It was found in more than 23% of retail chicken meat samples from Fukuoka Prefecture, Kyushu, Japan [1], and human salmonellosis cases caused by *S.* Infantis-contaminated chicken meat are relatively frequent in Kyushu [4, 5]. *S.* Infantis is also the dominant serovar in broiler farms in western Japan [6], although why and how it became the dominant serovar remains unresolved.

We hypothesised that *S.* Infantis may infect susceptible chickens at a higher frequency than other serovars, perhaps because of a higher basic reproductive rate in chickens [7]. However, little is known about the issue. Several studies have administered multiple *Salmonella* serovars at different intervals (1 day or more) in an attempt to understand the dynamics of infection [8, 9], but simultaneous administration of multiple *Salmonella* serovars is rare. Therefore, in the current study, we simultaneously infected 1-day-old chicks with *S.* Infantis and *S.* Typhimurium, and then housed the infected birds with non-infected chicks. The aim of the study was to determine whether *S*. Infantis more frequently passes from infected to non-infected chicks than *S*. Typhimurium.

## Methods

### *Salmonella* strains and chickens

*S*. Infantis strains 200–1, 1582 and 1596, isolated in 1995, 2005 and 2004, respectively, from chicken meat and broilers in western Japan, were used in the current study. All three strains belonged to the most dominant genotype, pulsed-field profile 4, as determined by pulsed-field gel electrophoresis analysis [10]. The three *S.* Typhimurium strains, 586, R6 and R38, were isolated from beef and humans in 2005, 1999 and 1999, respectively. All strains were stored at −80 °C.

Specific-pathogen-free (SPF) layer chickens (L-M line) were purchased from Nisseiken (Oume, Japan). At 0 days old, chicks were transported from Tokyo to Dazaifu by plane and car. Radiation-sterilised food (Funabashi Farm Co., Funabashi, Japan) and tap water were provided ad libitum, and sterilised bedding (Oriental Yeast Co., Tokyo, Japan) was used. Two to four birds were housed in each sterilised cage (Allentown, Allentown, NJ, USA), and cages were placed in a low-atmospheric-pressure caring apparatus (−10 hPa compared with room atmospheric pressure), which allowed for adjustments to temperature and humidity (Natsume Sesakusyo Co., Tokyo, Japan). Chicks were transferred to freshly autoclaved cages every 2 days during the experimental period. The temperature and humidity were initially set at 33 °C and 75%, respectively. These were decreased by 0.5 °C and 0.5%, respectively, per day, to achieve final conditions of 28.5 °C and 71.5%, respectively. The animal room was controlled with a 12 h light/dark cycle.

### *Salmonella* administration

Frozen (−80 °C) aliquots of each of the *Salmonella* strain stocks were inoculated into 3-ml volumes of Luria-Bertani (LB) broth (Becton Dickinson, Franklin Lakes, NJ, USA) and incubated with continuous shaking at 35 °C for about 18 h. The overnight bacterial cultures were then diluted with LB broth heated to 42 °C. The dilution volumes were determined by a preliminary dose-finding experiment (data not shown). Equal volumes of the three cultures of each serovar were mixed, and a 0.3-ml aliquot of the pooled cocktail of *S*. Typhimurium was administered into the crop of eight 1-day-old chicks using syringes with gavage needles. A 0.3-ml aliquot of *S.* Infantis cocktail was then immediately administered to the same chicks. Bacterial cell counts were carried out for each of the cocktails following administration, and showed that the 0.3-ml aliquots of *S.* Typhimurium and *S.* Infantis contained 2.7 × 10^6^ and 3.1 × 10^6^ colony-forming units, respectively.

### Caging design

**Fig. 1:**
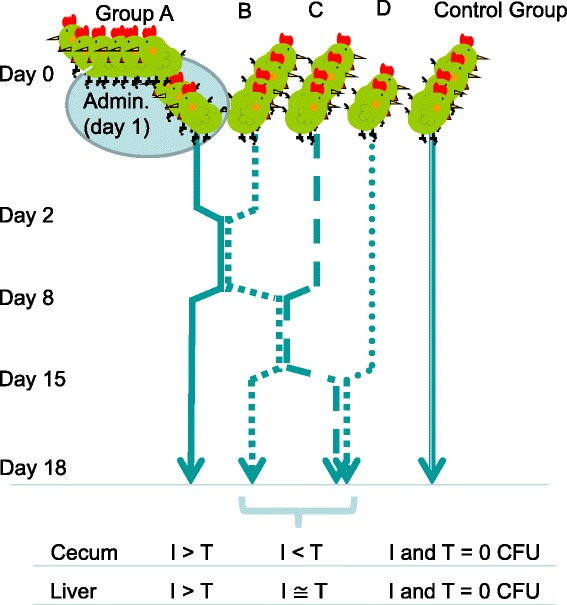
Experimental procedure for *Salmonella* administration. Eight 1-day-old birds were simultaneously administered with *Salmonella enterica* subspecies *enterica* serovar Infantis and *S.* Typhimurium (Group **a**), and then housed in the same cage as four non-infected birds (Group **b**) on days 2–8. Group **b** birds were then caged with Group **c** birds (four non-infected birds) on days 8–15. Group **c** birds were then housed with a final group of two non-infected birds (Group **d**) for days 15–18. I and T denote *S.* Infantis and *S*. Typhimurium, respectively. Five of eight birds were sacrificed at day 5 and examined using histopathology and immunohistochemistry

Figure 1 shows the caging schedule of the inoculated birds (seeder birds) with the non-infected birds (recipients). On day 2 post-inoculation (2 days old), the seeder birds (Group A, *n* = 8) were caged with the first group of recipients (Group B, *n* = 4). On day 8, Group B was caged with the second group of recipients (Group C, *n* = 4). Group C was then caged with the third recipient group (Group D, *n* = 2) on day 15. Control group birds (*n* = 4) were caged by themselves without any exposure to the *Salmonella* strains. All experimental animals were sacrificed by exsanguination under carbon dioxide gas-anaesthesia at day 18.

**Fig. 2:**
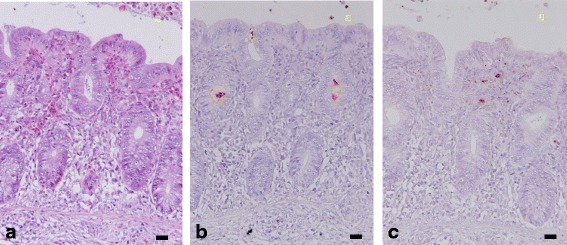
Immunostaining of cecal contents from chicks on day 5 post simultaneous administration of *Salmonella enterica* subspecies *enterica* serovar Infantis and *S.* Typhimurium. (**a**) Haematoxylin and eosin staining showing infiltration of a number of heterophils into the epithelial layer and cecal lamina propria. (**b**) Immuno-positive antigens against *Salmonella* serovar O7 were detected in cecal crypts. (**c**) Immuno-positive antigens against *Salmonella* serovar O4 were detected in the cecal lamina propria. White bars indicate 10 μm

### Enumeration of *Salmonella* from chick samples

Bowels and livers were dissected from the euthanised animals and then minced using sterilised scissors. The minced samples were then homogenised with 9 volumes of sterile saline using a Stomacher paddle blender (Seward, Worthing, UK). Ten-fold serial dilutions of the homogenised solutions were carried out, and 0.1 ml of each dilution was plated on *Salmonella*-*Shigella* (SS) agar (Eiken Chemical Co., Tokyo, Japan) in duplicate and incubated at 35 °C. SS agar was used on the basis of a preliminary agar selection test that showed similar growth support for both serovars. Following incubation for 2 days, *Salmonella* colonies were counted. Thirty isolates from each sample were identified as *S.* Typhimurium or *S.* Infantis using somatic (O) antisera O4 and O7, respectively (Denka Seiken Co., Tokyo, Japan). Statistical analyses were carried out using the chi-square test.

### Histopathology and immunohistochemistry

Five chickens from Group A were sacrificed at day 5 and examined using histopathology and immunohistochemistry. Chicken bowels were fixed with 20% formalin, and embedded in paraffin wax. Sections (3–4 μm thick) were then cut and stained with haematoxylin and eosin. Sections of the cecum, rectum and bursa of Fabricius were used for the detection of *Salmonella* serovar O4- and O7-group antigens. Sections were immunostained using the streptavidin-biotin-peroxidase conjugate (SAB) method, as previously described [11]. Controls for the SAB method were performed by omitting the primary antisera.

## Results

### Colonisation of chicks

Both *Salmonella* serovars were isolated from all samples collected from all birds, except for the control group. Levels of *S.* Infantis colonisation were significantly greater than those of *S.* Typhimurium in the bowel samples of Group A birds (*P* < 0.001) (Table 1). In contrast, the bowel samples of recipient birds (Groups B–D) showed significantly higher levels of *S.* Typhimurium colonisation compared with those of *S.* Infantis (*P* < 0.001; chi-square test). *S.* Infantis was also significantly more prevalent than *S.* Typhimurium in the liver samples of Group A birds (*P* < 0.001), whereas there was no difference in colonisation rates between the serovars in any of the liver samples from recipient birds (Groups B–D).

**Table 1:** *Salmonella enterica* subsp. *enterica* serovar (*S.*) Infantis and *S.*Typhimurium isolation rates at 18 days post administration of 1-day-old chicks

Organ	Group	Number of chicks	*Salmonella* colony count Mean ± SD (CFU / g)	*S*. Infantis %	Description
Bowels	A	3	1.6 × 10^7^ ± 0.6 × 10^7^	69 ± 11%	More *S*. Infantis was isolated^*^
	B	4	8.5 × 10^6^ ± 3.5 × 10^6^	40 ± 7%	More *S.* Typhimurium was isolated^*^
	C	4	1.4 × 10^7^ ± 0.8 × 10^7^	49 ± 13%	More *S.* Typhimurium was isolated^*^
	D	2	9.7 × 10^6^ ± 1.8 × 10^6^	44 ± 3%	More *S.* Typhimurium was isolated^*^
	Control	4	Not isolated		
Liver	A	3	2.6 × 10^4^ ± 3.3 × 10^4^	62 ± 18%	More *S*. Infantis was isolated^*^
	B	4	9.9 × 10^3^ ± 5.5 × 10^3^	68 ± 13%	No difference
	C	4	1.4 × 10^4^ ± 1.9 × 10^4^	53 ± 28%	No difference
	D	2	2.2 × 10^4^ ± 3.0 × 10^4^	35 ± 7%	No difference
	Control	4	Not isolated		

^*^ *P* < 0.001

Mean body weights (in g) at day 1 were as follows: Group A, 36.8 ± 4.0; Group B, 41.3 ± 1.2; Group C, 38.1 ± 1.4; Group D, 46.0 ± 0; control group, 41.3 ± 4.4. Mean body weights (in g) at day 18 were as follows: Group A, 154.5 ± 7.3; Group B, 177.3 ± 16.2; Group C, 167.9 ± 4.5; Group D, 207.5 ± 1.5; control group, 177.8 ± 12.4.

### Histopathology

Although there were no macroscopic lesions observed in the intestines of chicks administered with both *Salmonella* serovars, a number of instances of heterophil infiltration were observed in the epithelial layer and lamina propria of the cecum (Fig. 2a) and rectum. The lymphoid follicles of the bursa of Fabricius also had a “starry-sky” appearance.

### Immunohistochemistry

Several *Salmonella* serovar O4 antigens, indicating *S.* Typhimurium, and O7 antigens, indicating *S.* Infantis, were detected in cecal and rectal contents using immunohistochemistry. Although there were no *Salmonella* serovar O7 antigens in the parenchyma of the cecum (Fig. 2b), rectum or bursa of Fabricius, O7 immuno-positive signals were detected in cecal and rectal crypts. In addition, immuno-positive signals of *Salmonella* serovar O4 were detected in the lamina propria of the cecum (Fig. 2c) and rectum, as well as in lymphoid follicles of the bursa of Fabricius.

## Discussion

This study produced three main findings. First, no difference was observed in basic reproductive rates between the two serovars. Second, neither of the two serovars completely excluded the other, despite their competitive administration. Finally, *S.* Infantis invasion rates of the lamina propria of the cecum and rectum were lower than those of *S.* Typhimurium, even in the inoculated birds (Group A).

The findings of the current study, together with previous data, may explain the dominance of *S.* Infantis in chicken meat. A study in which heterologous serovars of *Salmonella* were administrated to chicks at different intervals showed that the first strain to be inoculated inhibited the colonisation of the subsequent strains [9]. However, using simultaneous administration, we observed that the heterologous strains never inhibited each other in the inoculated chicks. Together, these findings suggest that the predominant *Salmonella* strain or serovar in a given environment (e.g. farm) may infect chicks and then inhibit colonisation by other strains or serovars. Subsequently, one dominant strain or serovar continuously maintains a higher colonisation rate in those chicken flocks compared with other strains or serovars. This may explain why *S.* Infantis is the dominant serovar in chicken meat in Japan.

Variations in the susceptibility of different chicken lines to *Salmonella* infection were reported in the middle of the twentieth century [12]. More recently, Leveque et al. (2003) reported differences in resistance to *S.* Typhimurium infection between chicken lines resulting from allelic variation in Toll-like receptor 4 [13]. Hu et al. (1997) also reported differences in *Salmonella* susceptibility among chicken lines based on *Nramp1* (natural resistance-associated macrophage protein 1) and *Tnc* (a locus closely linked to *Lps*) variations [14]. Microbiota diversity in chicks can also affect susceptibility to infection [15]. However, little is known about differences in susceptibility to simultaneous inoculation of multiple *Salmonella* serovars in any chicken line. Therefore, while differences between chicken lines may affect susceptibility to *Salmonella* infection, in the current study, we focused on simultaneous infection with multiple *Salmonella* serovars. It would be interesting to carry out the same experiment in different chicken lines in the future to determine the effects of chicken line on susceptibility to simultaneous infection with multiple *Salmonella* serovars.

The simultaneous administration approach used in the current study produced different results from those described previously using individual administration of different *Salmonella* serovars [16]. Berndt et al. [16] reported that *S.* Infantis exhibited significantly lower invasion rates in the liver compared with *S.* Typhimurium after individual administration. In the present study, however, no differences were observed in the invasion rates of the liver between the two serovars. It is noteworthy that the two serovars never completely excluded each other in the liver after competitive administration. Non-detection of *S.* Infantis in the cecal lamina propria using immunohistochemistry may be the result of using sections from 5-day-old chicks. *S.* Infantis is less invasive of the cecal lamina propria at 5 days post-administration compared with at days 2 and 3 post-administration [9]. Moreover, a reduced ability to invade the cecal mucosa by *S.* Infantis compared with *S.* Typhimurium is consistent with the report by Berndt et al. [16].

## Conclusion

The basic reproductive rates in chicks do not appear to differ between *S.* Infantis and *S.* Typhimurium. Moreover, neither of the serovars displayed a superior ability to colonise the chick bowel in comparison with the other. Therefore, the quantitative domination of *S.* Infantis in chicks, and the associated inhibition of subsequent colonisation by other *Salmonella* strains, may explain why *S.* Infantis is the predominant *Salmonella* serovar in chickens and chicken meat in Japan.

## Abbreviations

LB: Luria-bertani
*S*.: *Salmonella enterica* subspecies *enterica*
SAB: Streptavidin-biotin-peroxidase conjugate
SPF: Specific-pathogen-free
SS: *Salmonella*-*Shigella*
